# Supplementary figures and images for: Antisense Oligonucleotides against Let-7 Enhance the Therapeutic Potential of Mesenchymal Stromal Cells
Source: Int J Mol Sci. 2023 May 12;24(10):8639. doi: 10.3390/ijms24108639 (PMC10218563; doi:10.3390/ijms24108639)

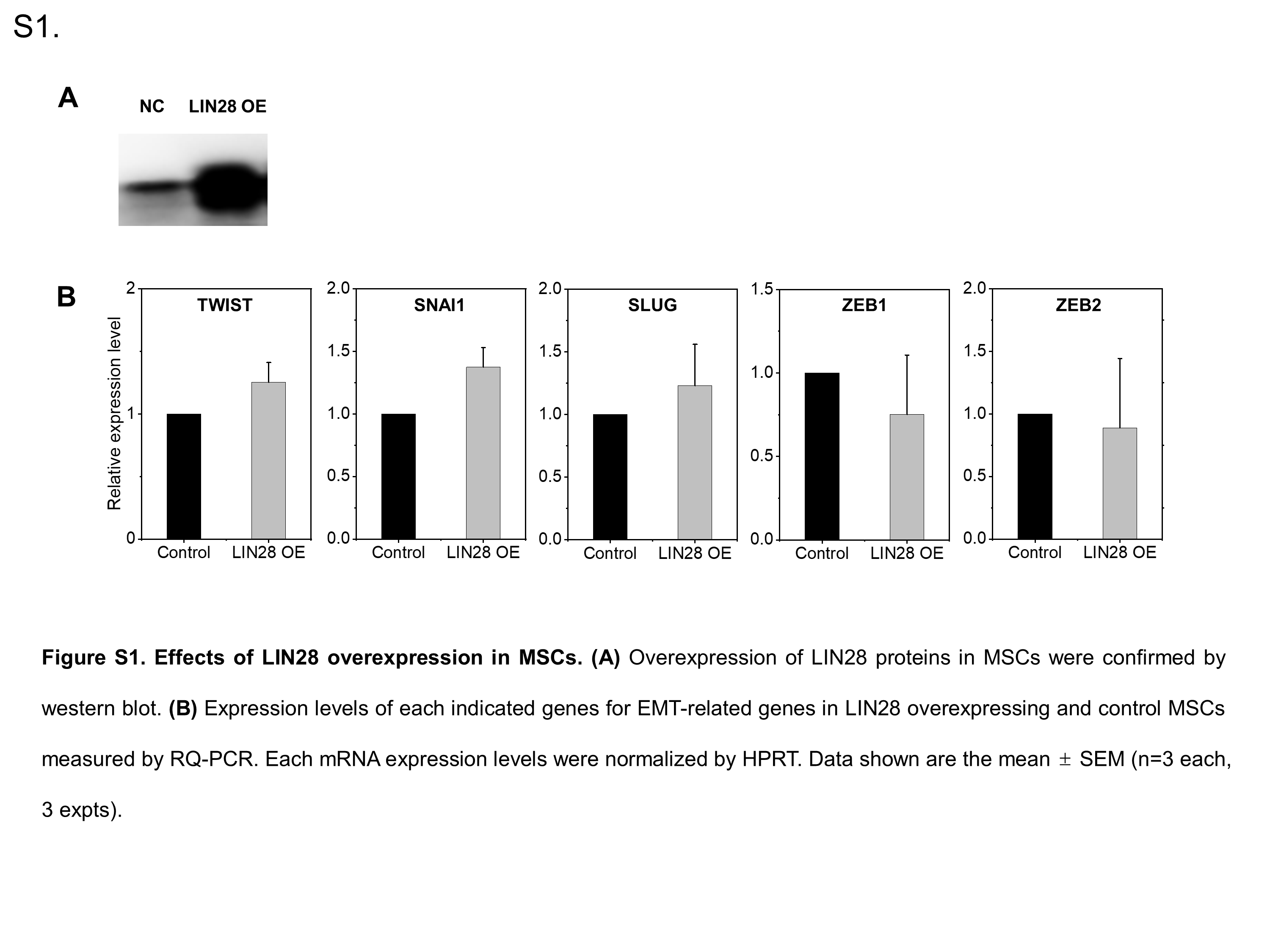

Supplement: Supplementary file 1 [file ijms-24-08639-s001.zip › Figure S1-OH.TIF]

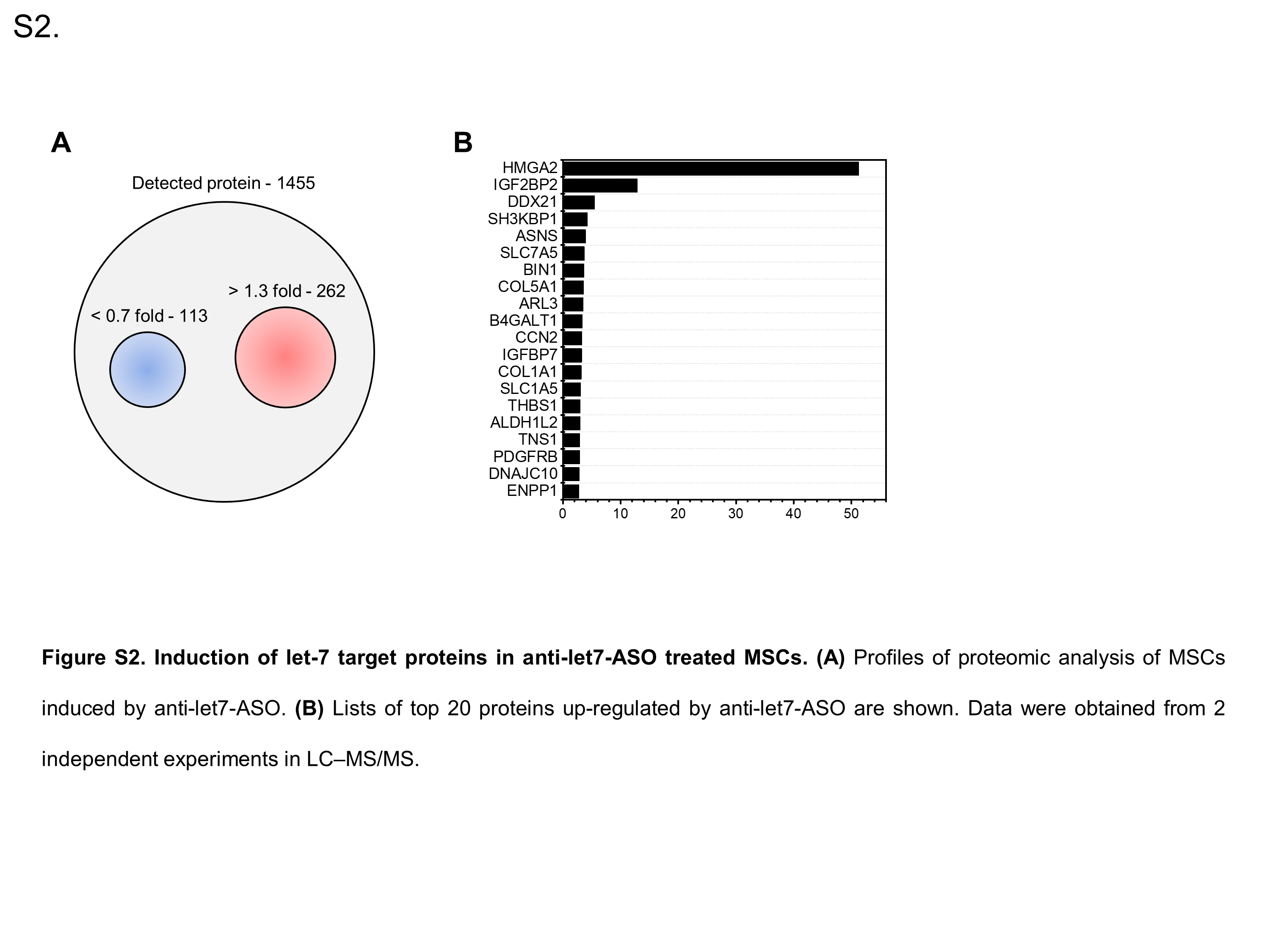

Supplement: Supplementary file 1 [file ijms-24-08639-s001.zip › Figure S2-OH.TIF]

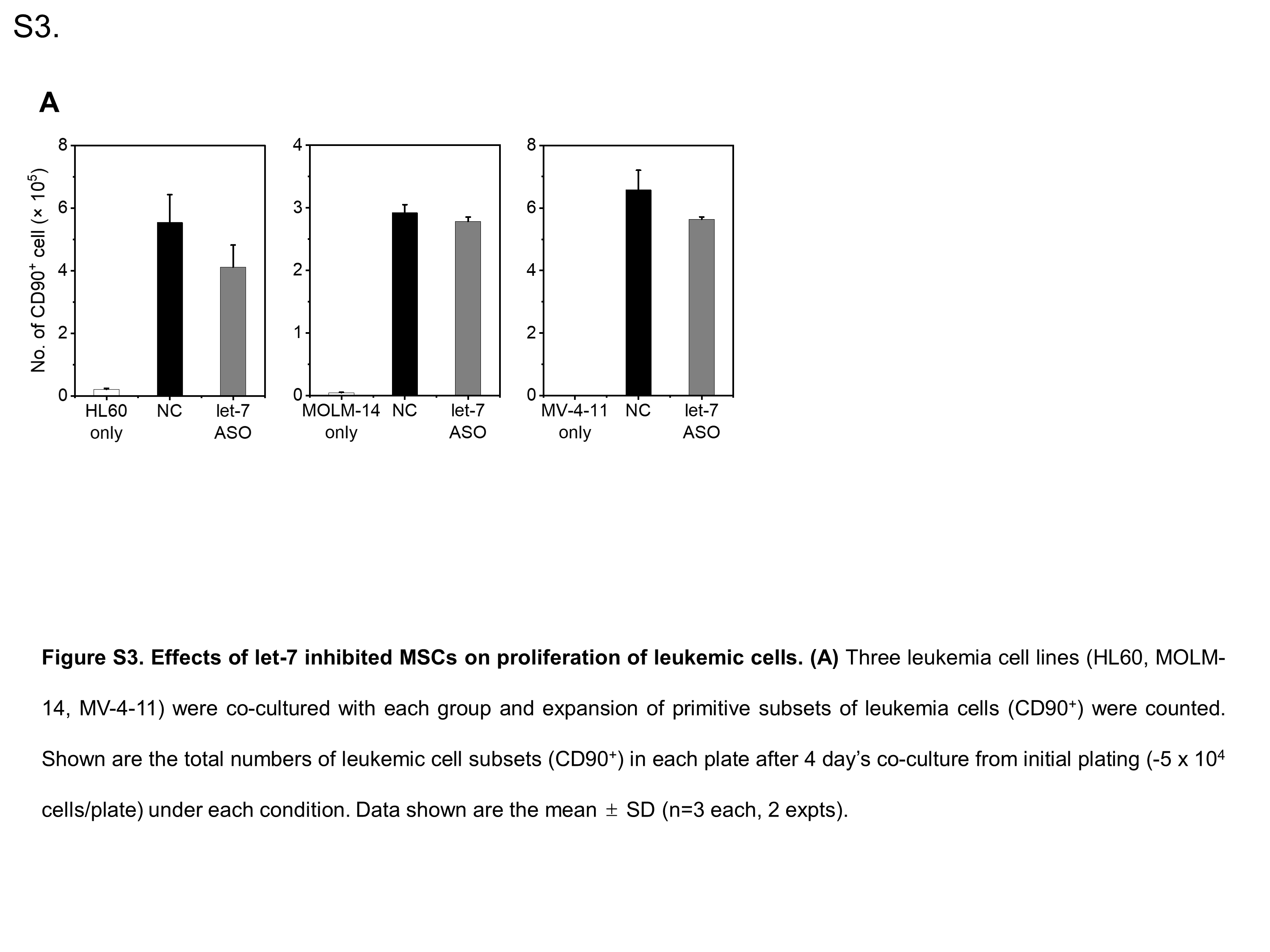

Supplement: Supplementary file 1 [file ijms-24-08639-s001.zip › Figure S3-OH.TIF]

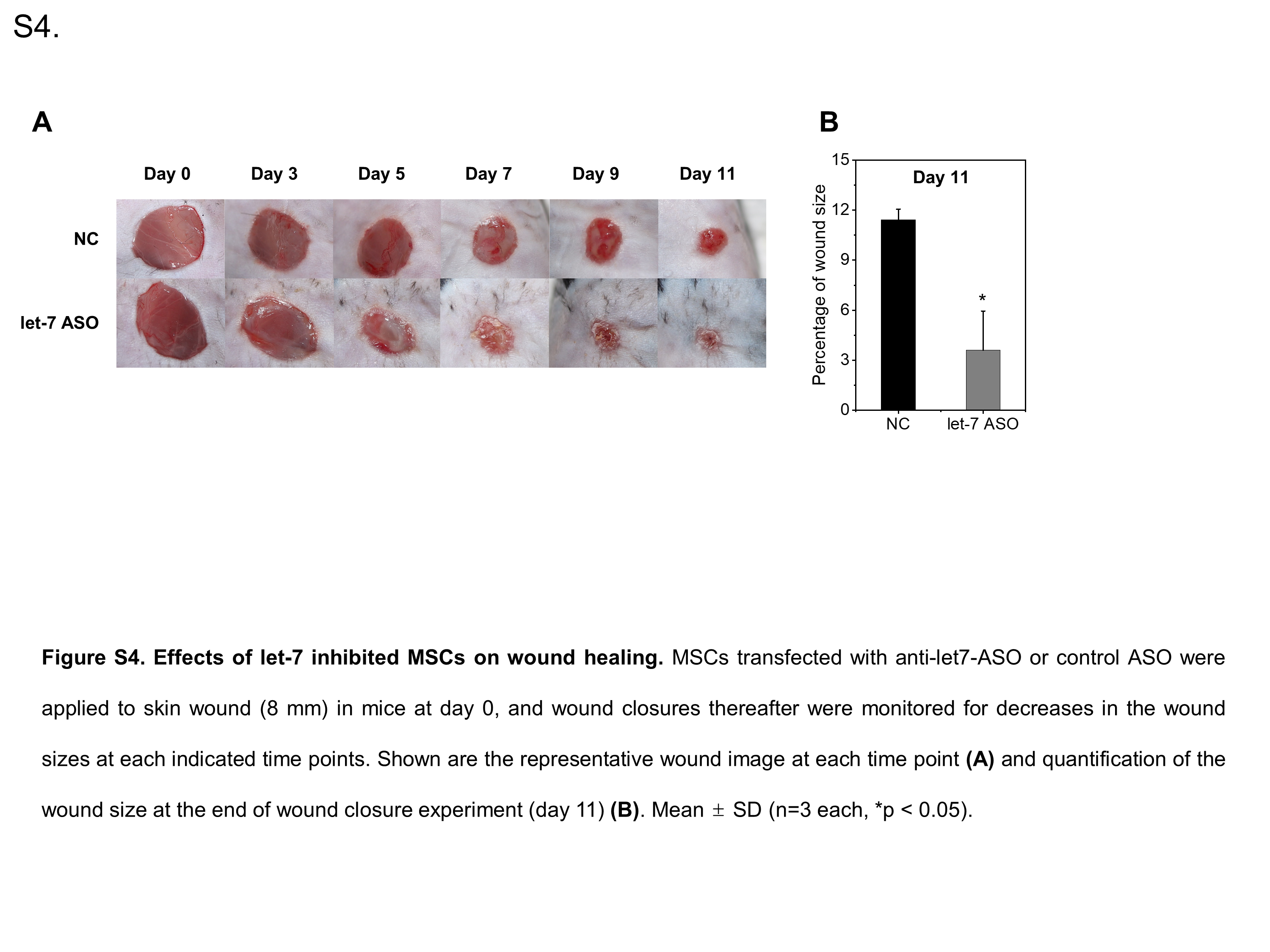

Supplement: Supplementary file 1 [file ijms-24-08639-s001.zip › Figure S4-OH.TIF]

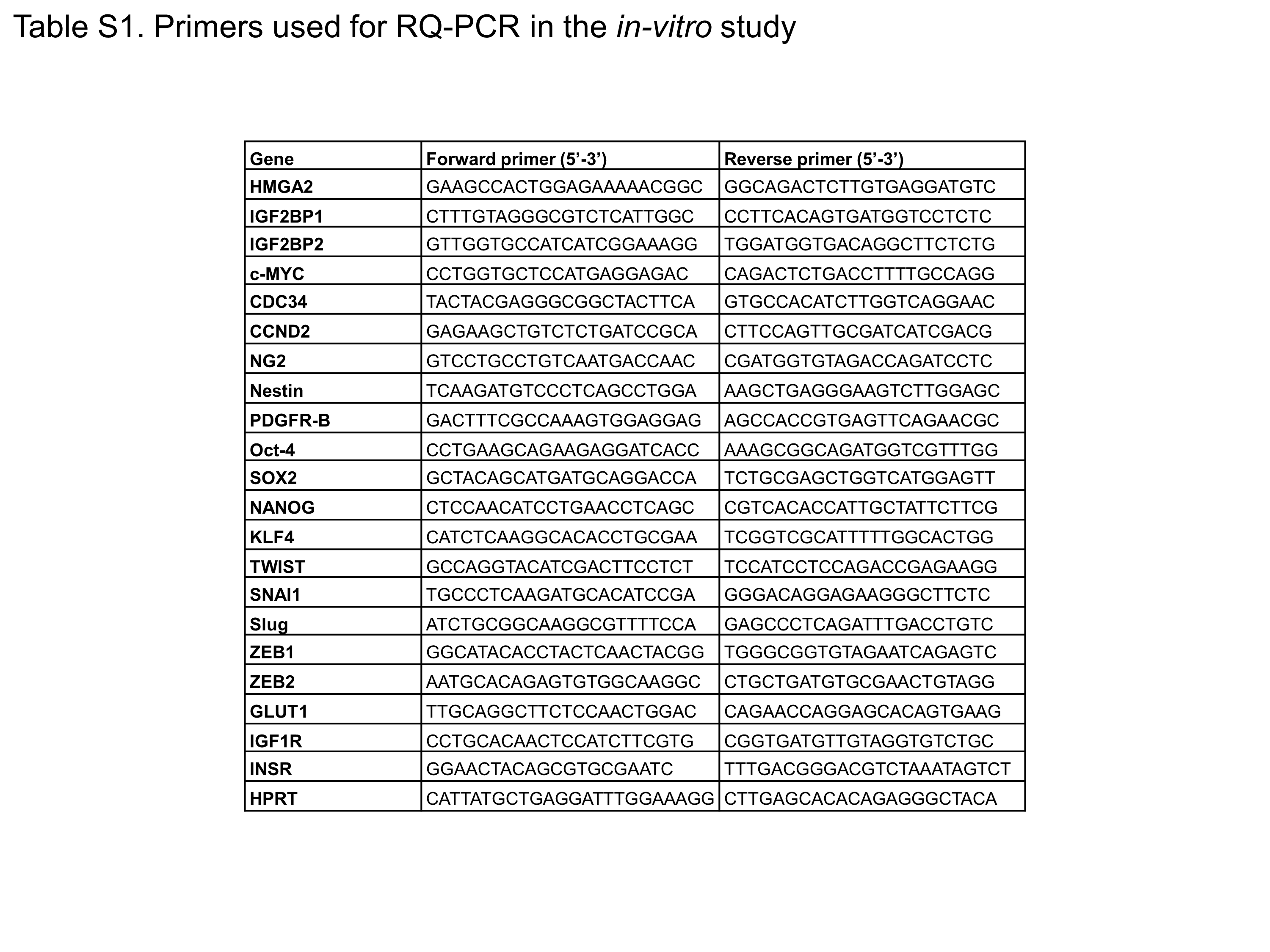

Supplement: Supplementary file 1 [file ijms-24-08639-s001.zip › Figure S5-OH.TIF]
